# Enhancing Patient Education for Colonoscopy Preparation: Strategies, Tools, and Best Practices

**DOI:** 10.3390/jcm14124375

**Published:** 2025-06-19

**Authors:** Roba Ganayem, Osama Alamour, Daniel L. Cohen, Nour Ealiwa, Naim Abu-Freha

**Affiliations:** 1 Faculty of Health Sciences, Ben Gurion University of the Negev, Beer-Sheva 84101, Israel; robaga311@gmail.com (R.G.); ealiwa@post.bgu.ac.il (N.E.); 2 Department of Emergency Medicine, Ben Gurion University of the Negev, Beer-Sheva 84101, Israel; osamaalamour28@gmail.com; 3 Gonczarowski Family Institute of Gastroenterology and Liver Diseases, Shamir Medical Center Assaf Harofeh, Tzrifin 6997801, Israel; docdannycohen@yahoo.com; 4 Institute of Gastroenterology, Assuta Beer Sheva, Assuta Medical Centers, Beer-Sheva 8489507, Israel; 5 Institute of Gastroenterology and Hepatology, Soroka University Medical Center, Beer-Sheva 84101, Israel

**Keywords:** colonoscopy, bowel preparation, short message service (SMS), visual aids, instructional videos, verbal communication, telephone support, mobile apps, virtual reality

## Abstract

**Background**: Colonoscopy is an important and essential diagnostic and screening tool for colorectal cancer and other pathologies in the colon. High-quality bowel preparation (BP) is a key quality measure of colonoscopy and is critical for maximizing its effectiveness, including enhancing adenoma detection rates. However, inadequate bowel preparation (IBP) remains a frequent challenge and is influenced by multiple factors. This review aims to summarize and evaluate educational and technological interventions implemented before colonoscopy to improve BP quality. **Methods**: The methodology comprised a structured narrative review of studies published in English, including randomized controlled trials, prospective studies, observational cohorts, and meta-analyses. Interventions were categorized by their delivery mode and impact on BP adequacy. Interventions included written materials, internet-based education modules, short message service (SMS) reminders, visual aids, instructional videos, verbal communication, telephone support, smartphone applications, and virtual reality (VR) platforms. **Results**: Most studies reported significant improvements in BP quality with enhanced patient education, particularly with the use of instructional videos and smartphone applications. Verbal communication and telephone support also demonstrated positive outcomes but were limited by resource availability. VR represents a promising emerging technology, though its implementation remains costly and complex. **Conclusions**: Enhanced educational interventions are proven methods to optimize BP quality. The selection of an appropriate modality should consider patient characteristics, technological accessibility, and institutional resources. Personalized strategies targeting high-risk populations can further reduce IBP rates and improve overall colonoscopy outcomes.

## 1. Introduction

Colonoscopy is an important diagnostic modality for common indications such as screening for colorectal cancer (CRC), positive fecal occult blood testing, and symptoms related to the gastrointestinal tract. CRC is the third most common cancer and the second most common cause of cancer mortality worldwide [1]. Detection of polyps, cancer, and other pathologies is an important part of the colonoscopy and requires adequate bowel preparation (BP). Thus, BP quality is a key performance measure of colonoscopy [2]. The need for BP in colonoscopy is one factor that negatively affects patients’ willingness to undergo CRC screening via colonoscopy [3,4]. In addition, adequate BP is necessary to perform a high-quality colonoscopy safely. Lastly, adequate BP facilitates the detection of colonic lesions, with the level of cleanliness and amount of residual stool crucially impacting the adenoma detection rate (ADR) and the detection of advanced adenomas [5,6].

Numerous major risk factors have been reported for inadequate BP, including demographic factors (male gender and older age), comorbidities (history of stroke or dementia and poorly controlled diabetes mellitus), medication use (opioids, tricyclic antidepressants, calcium channel blockers, and GLP1 agonists), and a body mass index (BMI) of more than 25 [7,8,9,10,11]. Additionally, other risk factors are related to poor compliance with the BP protocol, such as younger age, poor patient activation, minority race, lower socioeconomic status, lower level of education, poor health literacy, and a language barrier [12,13,14].

There are several methods used for the assessment of BP, with the Aronchick scale, the Ottawa Bowel Preparation Scale (OBPS), and the Boston Bowel Preparation Scale (BBPS) being the most commonly used tools [15,16,17,18]. These scales are used in colonoscopy studies to assess the quality of large bowel cleansing, and they report the quality of the large bowel in different ways. As such, it is difficult to make direct comparisons between studies that use different scoring systems.

To date, the use of technology in healthcare is rapidly expanding, revolutionizing the way medical services are delivered and accessed. Among the various technological innovations, the use of smartphones has become particularly significant due to their wide availability, portability, and user-friendly applications. These devices are increasingly being utilized in various aspects of healthcare, including patient education, remote monitoring, telemedicine consultations, chronic disease management, and enhancing patient compliance with treatment protocols. In the context of procedural preparation, such as bowel preparation before colonoscopy, smartphones offer an effective platform for delivering educational materials, sending reminders, and providing interactive guidance to improve patient outcomes. The integration of smartphone-based interventions represents a promising advancement toward more personalized, accessible, and efficient healthcare services worldwide.

Furthermore, reinforced education plays an important role in improving BP quality, increasing the ADR, decreasing the insertion and withdrawal time, and preventing adverse events [19]. Therefore, it is essential to provide instructions in both written and verbal formats as recommended by international guidelines for the preparation process [20,21]. Moreover, enhancing BP is recommended by the European Society of Gastrointestinal Endoscopy (ESGE) [21]. In a comprehensive systematic review and meta-analysis evaluating the association between educational interventions and colonoscopy quality indicators, educational interventions were associated with improvements in both the total and proximal ADR, with an increased withdrawal time, and without affecting the cecal intubation rate [22].

The aim of the present review is to summarize interventions reported in the literature that have been shown to improve BP quality while describing the different tools used and their effects on BP and other outcomes.

## 2. Materials and Methods

In this narrative review, a structured search of the English-language literature was conducted, focusing on studies published up to December 2024. The review included randomized controlled trials, prospective studies, observational studies, and meta-analyses that evaluated educational and technological interventions for enhancing BP quality. The interventions were categorized based on their mode of delivery, including written materials, internet-based education modules, short message service (SMS) reminders, visual aids, instructional videos, verbal communication, telephone support, smartphone applications, and virtual reality (VR) technologies. For each intervention type, we summarized its definition, key features, advantages, disadvantages, and the findings of relevant studies. Special attention was given to the methodology used in each study, including the scoring systems for bowel preparation assessment (such as the BBPS and the OBPS) and relevant clinical outcomes, such as the adequacy of preparation, adenoma detection rate (ADR), and polyp detection rate (PDR). Studies were included if they provided comparative data evaluating the effectiveness of an intervention against standard care or another educational strategy. Interventions were also evaluated for their accessibility, patient engagement potential, personalization capabilities, and cost-effectiveness. Studies with a small number of patients were not included.

## 3. Results

### 3.1. Interventions for Improving BP

In this section, the different strategies for improving BP are presented and discussed, including a short definition of each strategy, and the studies investigated and published for the specific intervention strategy are presented and discussed. The most commonly investigated strategies for improving BP quality are presented in Table 1, and the studies for each specific intervention are summarized in Table 2.

### 3.2. Written Materials

Written booklets/instructions about the BP process are provided to patients before colonoscopy. The information includes instructions regarding diet, medications, and bowel preparation, along with other important information regarding the procedure. Patients need to read these instructions and follow them as described. At present, written material is considered the standard of care in different studies and is compared to other interventions [23]. There is no standardization with respect to the content of the written materials, which are prepared locally and according to the needs of the patient population. Written materials are available in the local languages of the patients of a specific endoscopy department. The advantages of written materials are their low price, safety, and low level of resource consumption, but, in general, their efficacy is lower than that of other interventions. Using written materials is considered a baseline for educating patients before colonoscopy, and most studies compared new interventions and strategies to these written materials.

### 3.3. Websites or Patient Portals

Websites and patient portals are designed to deliver interactive and structured educational content to patients scheduled for colonoscopy. Such websites or portals could support patients by offering comprehensive instructions, multimedia materials, personalized reminders, and interactive checklists to guide patients through the bowel preparation process.

In a prospective study that used an internet-based education module to teach patients about BP, 84.6% achieved a BBPS of ≥6, and 90.1% had an Ottawa score of ≥7, showing that internet-based patient education is a viable option [24]. In another study, patients who visited a website for colonoscopy preparation prior to their procedure were more likely to have adequate BP than those who did not. The mean BPPS was 9 for those who had heard about the website but had not visited it, 8 for those who had never heard about the website, and 8.5 for individuals who had visited the website, visitation to the website was associated with higher adequate BP with an OR of 10.55 (1.35–82.4) [25].

Using websites for BP education provides good accessibility and is convenient for patients who can access the information anytime, anywhere, and with repeated viewings. In addition, standardized information delivered with multimedia capabilities is a cost-effective method that can be used by an unlimited number of patients.

However, this platform has several disadvantages, including a lack of internet access for some patients and issues with health literacy, particularly in older adults or lower socioeconomic groups. Additionally, the lack of personal interaction and the inability to ask questions are disadvantages of this platform. Finally, technical issues such as broken links and outdated browsers may negatively impact users.

### 3.4. Short Message Service (SMS) Texts

Sending a short text message to patients can provide support and education regarding BP. In general, these messages are sent using an automated SMS text messaging system in the days leading up to the colonoscopy and include dietary recommendations, bowel cleansing instructions, and information about the medication used for the preparation. Sending text messages in the days before a colonoscopy provides information to the patient while also allowing the patient to receive answers to questions regarding the BP or colonoscopy procedure. Several studies have shown that using SMS messages leads to improvement in BP quality in terms of a higher BBSP score and a lower rate of insufficient BP, as well as higher ADRs and PDRs among patients via reinforcement of education [26,27,28]. In addition, a meta-analysis that included seven randomized controlled studies with a total of 5889 patients reported a statistically higher rate of adequate BP in SMS groups compared to the control group (81.7% vs. 75.7%, *p* < 0.01) [29]. However, another study found no significant increase in the rate of adequate BP with the use of SMS texts [30].

The main advantage of using SMS messages to enhance BP is their high accessibility since almost all individuals have mobile phones and can receive SMS messages. Other advantages include the low cost of sending SMS texts, the lack of need for complicated computer platforms, their easy integration into healthcare systems, and the lack of a burden on the staff. However, there are several disadvantages that should be mentioned, including that the limited texts available may not sufficiently explain complex instructions, the lack of an option to answer patients’ questions, and that patients must be able to read and understand the language of the SMS messages. Technical barriers such as incorrect phone numbers blocked messages, or full inboxes can also hinder the delivery of the messages. Moreover, some patients may be concerned about receiving sensitive medical information over unsecured SMS messages, while others may be overloaded with messages and may ignore or miss the texts.

### 3.5. Visual Aids (VAs)

VAs include infographics via visual step-by-step guides, including images of preparation steps, charts, and timelines, which help patients track their progress and stay on schedule. Back et al. reported improvements in BP quality using audiovisual (AV) aids on smartphones (139 individuals in the intervention group and 144 controls; mean BBPS of 7.53 vs. 6.29, *p* < 0.001), along with increasing rates of preparation adherence and patient satisfaction [31]. An additional study also reported improvement in BP quality using VA interventions [32], although two other studies reported no improvement in BP [33,34]. A systematic review and meta-analysis published in 2023 that included six studies (1755 subjects) demonstrated that visual booklets are useful in improving bowel preparation. Outpatient settings and preparations not containing PEG could also benefit more from booklets [35].

### 3.6. Instructional Videos

Instructional videos are short and clear videos explaining the BP process. Using visual explanations makes complex instructions easy to understand, helping to clarify diet restrictions, timing, and medication use for BP. The use of educational instructional videos has several advantages, including their user-friendly visual experience, consistently providing the same high-quality information, and eliminating variation that can occur in the case of verbal instructions. Moreover, using videos can save healthcare staff time.

Several studies have investigated the impact of instructional videos on BP quality. Ho et al. reported improved BBPS scores of 8.54 among 95 patients after the introduction of instructional videos compared to the pre-interventional periods (8.54 vs. 7.9, *p* = 0.0039) [36]. Another randomized study highlighted the improvement in BP quality associated with using this tool in a comparison of a group of patients who received education via a brief instructional video with a control group of non-video patients, who received written BP recommendations. The quality of BP was measured using the Ottawa scale, and patient satisfaction with the preparation was assessed using a questionnaire tool. The study found the quality of BP assessed via the OBPS was lower in the video intervention group compared to the non-video group (4 vs. 5, *p* = 0.0002), while no difference was found in terms of patient satisfaction [37].

Despite this, most other studies have reported that using educational videos can significantly enhance the quality of BP [38,39,40,41,42]. A meta-analysis included eight trials comparing 990 participants receiving a video intervention with 987 controls and confirmed that providing instructional videos had a positive impact on BP quality (overall risk ratio (RR) = 1.20, 95% CI: 1.14–1.25, *p* < 0.001) and lowered the total Ottawa scores (overall standard mean difference (SMD) = −0.66, 95% CI: −0.91 to −0.42, *p* < 0.001), but no difference regarding PDR was found [43]. However, two other studies failed to demonstrate an improvement in BP quality after a video intervention [44,45], although, in Walker et al.’s study, subgroup analysis according to race and age found an improvement among African Americans and individuals older than 65 years [45].

### 3.7. Verbal Communication

The verbal communication method refers to one-on-one counseling in which nurses or doctors explain the colonoscopy process and important aspects of BP during a clinic visit. An additional option includes group sessions in which educational workshops are provided pre-procedure for a group of patients at the same time. In general, direct communication with medical staff is focused on the diet, hydration, and timing of BP medication.

One study reported improvements in BP quality in the education group following 10 min of verbal communication from a physician and written materials compared to information received from a medical secretary (adequate bowel preparation BBPS ≥ 5 of 90.4% vs. 74.7%, *p* = 0.021) [46]. Other studies have also reported improvements in BP quality, including the studies by Shieh et al. (adequate BP of 97.4% vs. 80%, *p* = 0.01) [47] and Elvas et al. (62% vs. 35%, *p* < 0.001) [48]. In another prospective, randomized study, patients either received in-person nurse education (group 1), no education (group 2), or telephone education (group 3). Colonoscopy cancellation rates due to poor preparation occurred at a lower rate in group 1 compared to the other groups (4.39% vs. 26.3% vs. 15.3%, *p* = 0.005), and the education program improved patient compliance and satisfaction [49]. These studies collectively highlight the positive impact of verbal and healthcare-delivered education on BP quality, with enhanced understanding and improved compliance.

### 3.8. Telephone Support

Follow-up telephone calls to clarify instructions and answer questions are an additional interventional educational method for improving BP among people undergoing colonoscopy. In a study by Liu et al., patients either received a telephone call the day before their colonoscopy or no intervention [50]. The percentage with adequate BP increased from 70.3% in the control group to 81.6% in the intervention group (*p* < 0.001), with Ottawa scores of 3.0 vs. 4.9 (*p* < 0.001), respectively [50]. Moreover, the PDR was higher (38% vs. 24.7%, *p* < 0.001) and the noncompliance rate was lower (9.4% vs. 32.6%, *p* < 0.001) among patients who underwent the telephone intervention [50]. However, no significant differences were observed between the two groups with regard to willingness to undergo repeat bowel preparation (*p* = 0.409).

A meta-analysis evaluating the effect of telephone instructions on the quality of BP in patients undergoing colonoscopy included nine studies with 1923 patients receiving the intervention and 1913 controls. The rate of adequate BP was significantly higher in the telephone group, with a pooled relative risk of 1.17 (95% confidence interval (CI): 1.05–1.30, *p* < 0.01) and a pooled mean difference of 1.32 for the BBPS (95% CI: 0.15–2.49, *p* < 0.05), while the Ottawa Bowel Preparation Scale score was −1.93 (95% CI: −2.35 to −1.51, *p* < 0.01) [51]. In addition, the PDR was significantly higher in the telephone group than in the control group (RR = 1.58, 95% CI: 1.23–2.04, *p* < 0.01), although no significant difference was noted in the ADR between the groups (RR = 1.37, 95% CI: 0.97–1.94, *p* = 0.08) [51].

In a study involving 162 elderly patients (aged 65 and older), the patients were re-educated via telephone by a specific nurse 2 days before colonoscopy and compared to those in a control group who received education only on the day of the appointment. A higher adequate BP was found in the intervention group, achieving a rate of 83.1%, compared to 59.5% in the control group (*p* = 0.03) [52].

### 3.9. Smartphone Applications

Mobile health technologies are educational tools that have been developed to provide access to BP instructions for colonoscopy. Mobile apps can be used to increase access to evidence-based care, better inform patients of care, increase the use of evidence-based practices, and enhance care after formal treatment has concluded [53]. Moreover, this technology can be used to increase awareness and education before medical interventions. In the case of BP, such technology can be helpful for education regarding the different important aspects of BP.

Smartphone applications have been developed in different countries, and their impact on BP quality has been investigated. Kang et al. compared 387 subjects after smartphone application intervention to 383 controls and found that the rate of adequate BP increased in the intervention group (82.2% vs. 69.5%, *p* < 0.001) [54]. Wen et al. showed a higher BBPS in the intervention group of 477 subjects compared to 473 controls (7.5 ± 1.2 vs. 6.5 ± 1.2, *p* < 0.001) [55]. Other studies with smaller numbers of subjects have also demonstrated improved BP [31,56,57]. In addition, two meta-analyses reported improvements in the rates of adequate BP and higher cleansing scores after smartphone application interventions [58,59]. While most studies and meta-analyses reported improvements in BP, it is important to mention that two studies failed to show improvements after intervention with smartphone applications [60,61]. This may be due to differences in study design, app content and features, timing, and duration of app use, and outcome assessment bias.

### 3.10. Virtual Reality

Virtual reality (VR) is an emerging technology involving interactive experiences within a computer-generated environment, in which participants can see, hear, and sometimes interact with the environment, making them feel like they are inside a different world. In general, participants wear headsets that cover their eyes. VR can be used for BP instruction by displaying clean vs. poorly prepared bowels, demonstrating the diet and laxative preparation in a step-by-step manner, and familiarizing the patient with the colonoscopy procedure.

Several studies have investigated the impact of interventions using VR on BP quality. Chen et al. investigated the impact of VR on 173 subjects compared to 173 controls and found significant increases in the BBPS in the intervention group (7.61 vs. 7.04, *p* = 0.002) [62]. Compliance increased from 50.3% to 68.8% (*p* < 0.001), and the PDR also increased from 26.7% to 41.9% (*p* = 0.03) [62]. In a smaller study of 40 subjects and 40 controls, the rate of adequate BP was significantly higher in the VR group (97.5% vs. 77.5%, *p* = 0.014) [63].

**Table 2:** Studies for every strategy investigated.

	Internet-Based Education Module			Results
	Interventions	Intervention Group	Control Group	
**Internet-based education module**				
Trasolini et al., 2019, Canada [24]	Prospective study, internet-based education for bowel preparation	900 patients	No control group	84.6% adequate bowel based on a BBPS ≥ 6 90.1% based on Ottawa bowel (≤7) preparation
Jain et al., 2022, Canada [25]	Prospective, cross-sectional survey study using a website	88	130 aware but did not visit the website; 288 unaware of the website	BBPS of 8.5 for individuals visiting the website, 9 for those that had heard of the website but had not visited it, and 8 for those that had never heard about the website (*p* = 0.013) OR of 10.55 (1.35–82.4) for visiting the website and adequate BP
**Sending SMS messages**				
Lee et al., 2015, Korea [26]	Prospective, randomized, blinded	SMS group (n = 125) Telephone group (n = 126)	Control group (n = 137)	BBPS of 7.1 in the telephone group, 6.8 in the SMS group, and 6.3 in the control group No difference in the polyp and adenoma detection rates
Walter et al., 2019, Germany [27]	Prospective, randomized, blinded, multicenter	SMS group (n = 248)	Control group (n = 247)	Lower rate of insufficient BP in the SMS group (9% vs. 19%, *p* = 0.0013) BBPS of 7.4 in the SMS group compared to 6.5 in the control group (*p* < 0.001) Higher ADR in the SMS group
Wang et al., 2019, China [28]	Prospective, blinded, randomized, single center	SMS group (n = 128) WeChat group (n = 127)	Control group (n = 125)	BBPS score: SMS group: 6.44 WeChat group: 6.81 Control group: 5.78 (<0.001) Adequate bowel preparation BBPS score ≥ 6 was 86.7% in the SMS group, 89.8% in the WeChat group, and 66.4% in the control group
Li et al., 2022, China [29]	Meta-analysis, 7 RCTs	5889 patients		Adequate BP of 81.7% in the SMS group and 75.7% in the control group
Mahmud et al., 2021, USA [30]	Prospective, randomized, single center	SMS group (n = 364)	Control group (n = 386)	No significant difference in appointment attendance or bowel preparation quality SMS group: Automated series of 9 education or reminder text messages Control group: Usual care, including written instructions and nurse telephone call
**Visuals Aids**				
Back et al., 2018, Korea [31]	Prospective, endoscopist-blinded, randomized	139 individuals	144 controls	BBPS of 7.53 vs. 6.29 (*p* < 0.001) for visual aids Adequate preparation (96.5%) using visual aids compared to not using such aids (73.6%, *p* < 0.001)
Tae et al., 2012, Korea [32]	Prospective, blinded, randomized, controlled trial	102 patients in the intervention group with visual aids	103 controls	BBPS of 7.4 ± 1.9 in the intervention group vs. 6.1 ± 2.2
Calderwood et al., 2011, USA [33]	Prospective, single center, blinded, randomized	n = 492	n = 477	BBPS score ≥5 was similar in both groups (91% visual aid vs. 89% control, *p* = 0.43)
Guardiol-Arevalo et al., 2019, Spain [34]	Prospective, single-center, endoscopist-blinded, randomized controlled trial	n = 66	n = 70	Median BBPS of 7 (6–9) in the intervention group vs. 6 (5.7–9, *p* = 0.17)
Losurdo et al., 2023 [35]	Systematic review and meta-analysis	n = 857	n = 898	Higher adequate BP (OR = 2.31, 95% CI: 1.20–4.45, *p* = 0.01)
**Instructional Videos**				
Ho et al., 2019, USA [36]	Singel center, retrospective	n = 95	n = 91	BBPS of 8.54 in the video group compared to 7.9 in the non-interventional group (*p* = −0.0039)
Prakash et al., 2013, USA [37]	RCT with outpatients	n = 67	n = 66	Ottawa Bowel Preparation Quality Scale score of 4 in the video group and 5 in the non-video group (*p* = 0.0002)
Pillai et al., 2018 [38]	Prospective, blind study with inner-city patients (undereducated, minority, African American)	n = 56	n = 48	A significant increase in “excellent”-grade adequate bowel preparation quality of >23% and a significant decrease in “inadequate” bowel preparations of almost 50%
Archer et al., 2024, Ireland [39]	Prospective, blinded trial	n = 251	n = 258	Adequate BP in 86.1% vs. 79.1% (*p* < 0.005)
Hayat et al., 2016, USA [40]	Prospective study	n = 1251	n = 1279	Higher rates of satisfactory bowel preparation (92.3% vs. 87.4%, *p* < 0.001) Lower rates of needing a repeat colonoscopy (3.3% vs. 6.6%, *p* < 0.001)
Ding et al., 2024, Australia [41]	Retrospective study	n = 617	n = 795	Lower rate of inadequate BP compared to the control group (6.3% vs. 9.8%, *p* = 0.018)
Hsueh et al., 2014, Taiwan [42]	Prospective	n = 104	n = 114	Adequate bowel preparation according to the Aronchick scale (80.8% vs. 48.2%, *p* < 0.001)
Ye et al., 2020 [43]	Systematic review and meta-analysis (8 RCTs)	n = 990	n = 987	Educational video group had a significantly higher incidence of adequate BP No difference in PDR
Rice et al., 2016, USA [44]	Prospective randomized, blinded, with outpatients	n = 42	n = 50	Adequate BP: 74 in the video group and 68% in the control group (*p* = 0.54)
Walker et al., 2022, USA [45]	RCT	n = 111	n = 102	BBPS of 8 in the video group and 7.6 in the control group (*p* = 0.076), but significant differences in African Americans and people older than 65 years
**Verbal communication**				
Dikkanoglu et al., 2021, Turkey [46]	RCT with 10-min physician-delivered education	n = 73	n = 75	The rate of adequate BP (BBPS score ≥ 5) was 90.4% in the intervention group and 74.7% in the control group (*p* = 0.021)
Shieh et al., 2013, Taiwan [47]	RCT with 10-min physician-delivered education	n = 39	n = 60	The rate of adequate BP (BBPS score ≥ 5) was 97.4% in the intervention group and 80% in the control group (*p* = 0.01)
Elvas et al., 2017, Portugal [48]	RCT with personal instructions by a nurse	n = 113	n = 116	Adequate BP (Aronchick scale) rate of 62% in the intervention group and 35% in the control group (*p* < 0.001)
Abuksis et al., 2001, Israel [49]	RCT with nurse education	n = 91 nurse instructions n = 13 telephonic instructions	n = 38	Poor preparation rate of 4.38% in the education group compared to 26.3% in the control group and 15.38% in the telephone group
**Telephone Support**				
Liu et al., 2014 China [50]	RCT	n = 305	n = 300	Adequate BP rate of 81.6% in the intervention group and 70.3% in the control group (*p* < 0.001) Polyp detection rate of 38% vs. 24.7% (*p* < 0.001); Ottawa scores of 3 vs. 4.9 (*p* < 0.001); and non-compliance rate of 9.4% vs. 32.6% (*p* < 0.001)
He et al., 2023 [51]	Meta-analysis, 9 RCTs	n = 1923	n = 1913	Increased the rate of adequate BP, with a pooled relative risk of 1.17, a mean difference of 1.32 in BBPS (*p* < 0.01), an Ottawa score of 1.93 (*p* < 0.01), and a polyp detection rate of 0.08; the polyp detection rate increased significantly in the telephone group compared to the control group (RR = 1.58)
Hu et al., 2021 China [52]	RCT	In total, n = 162		A higher adequate BP: 83.1% vs. 59.5% (*p* = 0.03)
**Smartphone Applications**				
Kang et al., 2016, China [54]	RCT	n = 387	n = 383	Adequate BP rate of 82.2% of the intervention group compared to 69.5% in the control group (*p* < 0.001)
Wen et al., 2022, China [55].	RCT, multicenter	n = 477	n = 473	Higher BBPS of 7.5 ± 1.2 vs. 7.5 ± 1.3 vs. 6.5 ± 1.2 (*p* < 0.001)
Lorenzo-Zuniga et al., 2015, Spain [56]	RCT	n = 108	n = 152	Successful BP: 100% vs. 96.1% (*p* = 0.037)
Back et al., 2018, Korea [31]	RCT	n = 139	n = 144	Mean BBPS of 7.53 vs. 6.29 (*p* < 0.001); adequate BP in 96.5% vs. 73.6% (*p* < 0.001)
Cho et al., 2017, Korea [57]	Prospective study	n = 71	n = 71	BBPS score was higher in the intervention group (7.70 ± 1.1 vs. 7.24 ± 0.8, respectively; *p* = 0.007)
Desai et al., 2019 [58]	Systematic review and meta-analysis (6 RCTs)	n = 810	n = 855	Higher proportion of adequate BP (87.5% vs. 77.5%, pooled)
Bizri et al., 2021 [59]	Systematic review and meta-analysis (10 RCTs)			BBPS standardized mean difference (SMD) of 0.57, 95% CI: 0.37–0.77, I2 = 60% (*p* = 0.08), and Ottawa Bowel Preparation Scale (SMD −0.39, 95% CI: −0.59 to 0.19, I2 = 45%, *p* = 0.16)
Dao et al., 2023, Vietnam [60]	RCT	n = 256	n = 259	Proportion of poor preparation: 7.4% vs. 7.7% (*p* = 0.90) Median BBPS: 7.5 vs. 7 (*p* = 0.02); PDR: 23.4% vs. 23.2% (*p* = 0.94); ADR: 9 vs. 8.9% (*p* = 0.97) Higher adherence to instructions: 60.9% vs. 52.4% (*p* = 0.05)
Sharara et al., 2017, Lebanon [61].	RCT	n = 80	n = 80	Based on the Aronchick scale, adequate BP in 77.2% vs. 82.5 (*p* = 0.68) No difference in adherence (82.4% vs. 73.4%, *p* = 0.40)
**Virtual Reality**				
Chen et al., 2021, China [62]	RCT	n = 173	n = 173	Mean BBPS of 7.61 (1.65) vs, 7.04 (1.70) (*p* = 0.002) PDR of 41.9% vs. 26.7% (*p* = 0.003); ADR of 32.6% vs. 22.1% (*p* = 0.03) Higher compliance (68.8% vs. 50.3%, *p* < 0.001)
Gwag et al., 2024, Korea [63]	RCT	n = 40	n = 40	Adequate BP of 97.5% vs. 77.5% (*p* = 0.014)

## 4. Discussion

BP is an important quality measure for colonoscopy and impacts the success of the procedure. Still, a recently published meta-analysis that included 67 studies reported a proportion of IBP across the studies ranging from 5% to 67%, with a median of 26% [64]. Moreover, IBP increased the direct cost of colonoscopy by 12% to 22%, depending on the practice setting, due to the longer duration of the examination and the need for repeated procedures [65]. The European Society of Gastroenterology recommends an IBP rate of less than 10% [2]. As there are many conditions and risk factors that increase the rate of IBP [65], it seems that intervention is needed, and possibly mandatory, to decrease the rate of IBP. Hassan et al. proposed a predictive model based on patients’ demographics and comorbidities that could theoretically decrease the IBP rate from 33% to 13% in the case of 100% efficacy [66].

In this review, we collected and summarized information and data regarding the common methods and technologies for intervention to decrease the rate of IBP, including written materials (used in most endoscopy departments), internet-based education modules, SMS messaging, visual aids, instructional videos, verbal communication, telephone support, mobile apps, and virtual reality. While most studies showed an improvement in the rates of adequate BP with intervention, a few studies demonstrated no significant improvements using the same modality.

Interventions not only impact BP quality (OR 2.59, 95% CI: 2.09–3.19; *p* < 0.001) but also affect other outcomes such as increasing the ADR (OR = 1.35) and PDR (OR = 1.24), shorter insertion and withdrawal times, and increasing compliance with the preparation diet [19].

Considering all the positive effects mentioned above, along with the cost-effectiveness of these interventions, increases in patient satisfaction, recommendations from medical associations, and the advancement of emerging technologies and digital awareness, it appears increasingly important, and perhaps even necessary, to offer some of these additional interventions for patients scheduled for colonoscopy rather than relying solely on providing written materials.

### 4.1. Which Modality to Choose?

There are numerous factors that are important and helpful when choosing the type of intervention, including the following.

Characteristics of the target population such as age, digital literacy, and language and culture aspects. While older people may prefer simple methods such as verbal or telephone support, younger people may prefer smartphone apps, videos, or VR technology. Moreover, the materials provided need to be understandable and adopted culturally.

### 4.2. Institutional Resources and Budget

While written materials, SMS messages, and telephone calls are cheaper, using VR, smartphone apps, video production, and verbal communication are more expensive. Staffing availability is important for verbal communication (10-min meetings) and telephone education.

In summary, written materials should serve as a baseline service in every endoscopy unit; however, they are often insufficient when used alone. Internet-based modules are effective and appropriate for self-motivated, literate patients with internet access. Sending SMS texts is a low-cost strategy that enhances compliance and BP, ranging from simple appointment reminders to detailed BP instructions. Visual aids improve comprehension and are generally suitable for all patient groups, though certain populations may benefit more from this approach. Despite the lack of individualized interaction, instructional videos are among the most extensively studied methods, demonstrating significant improvements in BP. Personal communication with physicians, gastroenterologists, or nurses yields good outcomes by allowing for real-time questions, but it is time-consuming and often limited by resource constraints. Telephone support can be structured in various ways and has shown promise. Several studies have demonstrated the efficacy of smartphone applications in improving BP, offering high engagement and personalization. VR is an emerging tool with potential, but it remains costly and complex to implement at this time. Currently, the use of instructional videos or smartphone applications appears to be the most promising and accessible strategy. Additionally, personalized medicine should be considered when planning interventions, particularly focusing on patients at higher risk of inadequate BP such as male patients with constipation, cirrhosis, diabetes mellitus, hypertension, stroke, or dementia [64] or those using medications like opioids, tricyclic antidepressants [64], and newer agents such as glucagon-like peptide 1 receptor agonists [11].

### 4.3. Best Practices for Implementation of Interventions in Real-Life

All policymakers and directors of endoscopy units are encouraged to add any of the interventions mentioned according to their local population’s needs and the available resources, with specific consideration regarding the linguistic and cultural needs of the population. When implementing an intervention, the following points are important to consider.

- Provider engagement: Training healthcare providers on effective communication and standardized education protocols.
- Feedback mechanisms: Collecting and analyzing patient feedback. Adopting materials based on patient needs, with ongoing monitoring of the results of the intervention.
- Cultural and personalized adopted interventions: Incorporating cultural beliefs and practices. Availability of interpreters or culturally aligned educators.
- Continuous quality improvement: Monitoring preparation outcomes. Refining strategies through data-driven insights.

Lastly, this comprehensive synthesis provides practical insights for healthcare providers, endoscopy unit directors, and policymakers in selecting appropriate and evidence-based strategies to optimize bowel preparation outcomes.

There are several strengths to this present review. First, it provides a comprehensive and updated summary of a wide range of educational and technological interventions aimed at improving BP quality during colonoscopy. Second, it systematically categorizes interventions based on their mode of delivery and offers a structured and practical framework that can assist clinicians, endoscopy unit managers, and policymakers in decision-making. Third, the review highlights the advantages, disadvantages, and practical considerations of each intervention. However, several limitations should be mentioned. This review focused on English-language literature and, thus, non-English publications were not included, which may have introduced language bias and excluded potentially relevant studies. Second, the review is narrative in nature and not a systematic review or meta-analysis; thus, it did not include a formal risk of bias assessment for the individual studies or a pooled quantitative analysis. Third, the included studies varied widely in their design, patient populations, outcome measures, and definitions of adequate BP, making direct comparisons challenging. Fourth, variations in healthcare settings, resource availability, cultural differences, and patient populations across studies may limit the applicability of some interventions to all clinical environments.

## 5. Conclusions

Educational interventions prior to colonoscopy are needed to improve the quality of BP. Numerous interventions are available and have shown efficacy in improving BP quality. The appropriate intervention should be chosen based on local preferences, characteristics of the target population, and institutional resources and budget.

## Figures and Tables

**Table 1:** Interventions for improving bowel preparation.

		Description	Advantages	Disadvantages
1	Written materials	Written materials regarding the diet and medication for colonoscopy are given to the patient	Low price Practical Safe and unambiguous	Lower efficacy than other interventions
2	Internet-based education module	Website for bowel preparation, including diet and medication description according to the type of preparation and time	Accessibility and standardization of information delivery Multiple capabilities and personalization Cost-effective	Internet access could be limited in different regions Health literacy barriers Lack of personal interaction Language and cultural limitations Overreliance on technology
3	Short message service (SMS) texts	Sending short text messages to the patient regarding the preparation	High accessibility Low costs Easy implementation Improved compliance Timely reminders Reduced staff burden	Limited number of words Patients must be able to read the text language Technical barriers (incorrect phone numbers, blocked messages, or full inboxes can prevent delivery Privacy concerns Ignoring or missing SMS messages due to message overload No interaction or feedback
4	Visual aids	Using infographics via visual step-by-step guides, including images of preparation steps	Improve understanding Simplify complex information Overcome language barriers Enhance memory and recall, as people tend to remember visual information Increased engagement Cost-effective (simple and printed)	Limited details in the images Potential misinterpretation and misunderstanding of images Generic and not personalized Design quality
5	Instructional videos	Using videos to explain the bowel preparation process	Visual explanations Easy to understand high-quality information, eliminating variation in verbal instructions Saving healthcare staff time Use of different languages and culturally tailored versions	Specific groups could have limited access to the internet, smartphones, or skills to watch videos One-way communication, with no option for real-time questions May not address specific medical conditions
6	Verbal communication	Direct, face-to-face, telephone, or virtual meetings between the patient and a healthcare provider (nurse or gastroenterologist)	Personalized education Immediate clarification and answering of questions Build trust and reduce anxiety Early detection of any barriers (mobility problems, misunderstanding of timing, etc.) Encourage adherence	Time-consuming Intensive staff resources Inconsistency of the instructions provided by different providers Patient recall limitations Language barriers
7	Telephone support	Structured, proactive phone calls made by healthcare providers	Direct one-to-one communication tailored to patient specific needs Real-time clarifications Increase adherence Cost effective Build trust and reduce anxiety	Requires significant staff time Staff must coordinate calling times Patients may not answer an unknown number Information may vary between staff members Patients may forget what was discussed Privacy concern
8	Smartphone apps	Smartphone-based applications specifically designed to guide patients	Personalized guidance according to the patient’s characteristics Automatic reminders Using multimedia Convenient access Data collection including feedback Support trend of digital literacy	Barriers of technology access Requires initial investment (time, money, and technical support) Need continuous updating and technical support Dependence on internet and device functionality Privacy and security risks Variable quality of the apps (design and user-friendliness)
9	Virtual reality	Providing interactive experiences, VR aims to improve the quality of bowel cleansing, reduce patient anxiety, and increase overall satisfaction with the procedure	Better understanding Reduced anxiety Increased engagement and motivation	Limited accessibility High costs Technological barriers, challenge in using the equipment effectively A minority of the patients may experience motion sickness or discomfort

## Abbreviations

The following abbreviations are used in this manuscript:

ESGE	European Society of Gastrointestinal Endoscopy
PDR	Polyp detection rate
ADR	Adenoma detection rate

## References

[1] Sung H., Ferlay J., Siegel R.L., Laversanne M., Soerjomataram I., Jemal A., Bray F. (2021). Global cancer statistics 2020: GLOBOCAN estimates of incidence and mortality worldwide for 36 cancers in 185 countries. CA Cancer J. Clin..

[2] Kaminski M., Thomas-Gibson S., Bugajski M., Bretthauer M., Rees C., Dekker E., Hoff G., Jover R., Suchanek S., Ferlitsch M. (2017). Performance measures for lower gastrointestinal endoscopy: A European Society of Gastrointestinal Endoscopy (ESGE) quality improvement initiative. Endoscopy.

[3] Jones R.M., Devers K.J., Kuzel A.J., Woolf S.H. (2010). Patient-reported barriers to colorectal cancer screening: A mixed-methods analysis. Am. J. Prev. Med..

[4] Jover R., Zapater P., Polanía E., Bujanda L., Lanas A., Hermo J.A., COLONPREV Study Investigators (2013). Modifiable endoscopic factors that influence the adenoma detection rate in colorectal cancer screening colonoscopies. Gastrointest. Endosc..

[5] Guo R., Wang Y.-J., Liu M., Ge J., Zhang L.-Y., Ma L., Huang W.-Y., Zhai H.-H. (2019). The effect of quality of segmental bowel preparation on adenoma detection rate. BMC Gastroenterol..

[6] Sharma P., Burke C.A., Johnson D.A., Cash B.D. (2020). The importance of colonoscopy bowel preparation for the detection of colorectal lesions and colorectal cancer prevention. Endosc. Int. Open.

[7] Yee R., Manoharan S., Hall C., Hayashi A. (2015). Optimizing bowel preparation for colonoscopy: What are the predictors of an inadequate preparation?. Am. J. Surg..

[8] Romero R., Mahadeva S. (2013). Factors influencing quality of bowel preparation for colonoscopy. World J. Gastrointest. Endosc..

[9] Feng L., Guan J., Dong R., Zhao K., Zhang M., Xia S., Zhang Y., Chen L., Xiao F., Liao J. (2024). Risk factors for inadequate bowel preparation before colonoscopy: A meta-analysis. J. Evid.-Based Med..

[10] Mahmood S., Farooqui S., Madhoun M. (2018). Predictors of inadequate bowel preparation for colonoscopy: A systematic review and meta-analysis. Eur. J. Gastroenterol. Hepatol..

[11] Abu-Freha N., Yitzhak A., Shirin H., Nevo-Shor A., Abu-Jaffar J., Abu-Rafe S., Afianish Y., Cohen D.L., Bermont A. (2025). Glucagon-like peptide-1 receptor agonists significantly affect the quality of bowel preparation for colonoscopy. Endoscopy.

[12] King-Marshall E.C., Mueller N., Dailey A., Barnett T.E., George T.J., Sultan S., Curbow B. (2016). “It is just another test they want to do”: Patient and caregiver understanding of the colonoscopy procedure. Patient Educ. Couns..

[13] Cohen L.B. (2015). Advances in bowel preparation for colonoscopy. Gastrointest. Endosc. Clin..

[14] Austin K.L., Power E., Solarin I., Atkin W.S., Wardle J., Robb K.A. (2009). Perceived barriers to flexible sigmoidoscopy screening for colorectal cancer among UK ethnic minority groups: A qualitative study. J. Med. Screen..

[15] Kastenberg D., Bertiger G., Brogadir S. (2018). Bowel preparation quality scales for colonoscopy. World J. Gastroenterol..

[16] Sawhney M.S. (2015). Bowel preparation for colonoscopy: Assessing and improving quality. Gastroenterol. Endosc. News.

[17] Bechtold M.L., Mir F., Puli S.R., Nguyen D.L. (2016). Optimizing bowel preparation for colonoscopy: A guide to enhance quality of visualization. Ann. Gastroenterol. Q. Publ. Hell. Soc. Gastroenterol..

[18] Parmar R., Martel M., Rostom A., Barkun A.N. (2016). Validated scales for colon cleansing: A systematic review. Off. J. Am. Coll. Gastroenterol.|ACG.

[19] Guo X., Li X., Wang Z., Zhai J., Liu Q., Ding K., Pan Y. (2020). Reinforced education improves the quality of bowel preparation for colonoscopy: An updated meta-analysis of randomized controlled trials. PLoS ONE.

[20] Johnson D.A., Barkun A.N., Cohen L.B., Dominitz J.A., Kaltenbach T., Martel M., Rex D.K. (2014). Optimizing adequacy of bowel cleansing for colonoscopy: Recommendations from the US multi-society task force on colorectal cancer. Off. J. Am. Coll. Gastroenterol.|ACG.

[21] Hassan C., East J., Radaelli F., Spada C., Benamouzig R., Bisschops R., Dumonceau J.M. (2019). Bowel preparation for colonoscopy: European Society of Gastrointestinal Endoscopy (ESGE) guideline—Update 2019. Endoscopy.

[22] Causada-Calo N.S., Gonzalez-Moreno E.I., Bishay K., Shorr R., Dube C., Heitman S.J., Forbes N. (2020). Educational interventions are associated with improvements in colonoscopy quality indicators: A systematic review and meta-analysis. Endosc. Int. Open.

[23] Kurlander J.E., Sondhi A.R., Waljee A.K., Menees S.B., Connell C.M., Schoenfeld P.S., Saini S.D. (2016). How efficacious are patient education interventions to improve bowel preparation for colonoscopy? A systematic review. PLoS ONE.

[24] Trasolini R., Nap-Hill E., Suzuki M., Galorport C., Yonge J., Amar J., Enns R.A. (2019). Internet-based patient education prior to colonoscopy: Prospective, observational study of a single center’s implementation, with objective assessment of bowel preparation quality and patient satisfaction. J. Can. Assoc. Gastroenterol..

[25] Jain A., Jain R., Nugent Z., Solati Z., Davidson D., Shafer L.A., Restall G., Reynolds K., Singh H. (2022). Improving Colonoscopy Bowel Preparation and Reducing Patient Anxiety Through Recently Developed Online Information Resource: A Cross-sectional Study. J. Can. Assoc. Gastroenterol..

[26] Lee Y.J., Kim E.S., Choi J.H., Lee K.I., Park K.S., Cho K.B., Hwang J.S. (2015). Impact of reinforced education by telephone and short message service on the quality of bowel preparation: A randomized controlled study. Endoscopy.

[27] Walter B., Klare P., Strehle K., Aschenbeck J., Ludwig L., Dikopoulos N., von Delius S. (2019). Improving the quality and acceptance of colonoscopy preparation by reinforced patient education with short message service: Results from a randomized, multicenter study (PERICLES-II). Gastrointest. Endosc..

[28] Wang S.L., Wang Q., Yao J., Zhao S.B., Wang L.S., Li Z.S., Bai Y. (2019). Effect of WeChat and short message service on bowel preparation: An endoscopist-blinded, randomized controlled trial. Eur. J. Gastroenterol. Hepatol..

[29] Li P., He X., Dong J., Chen Y., Zhou Q. (2022). Reinforced education by short message service improves the quality of bowel preparation for colonoscopy. Int. J. Color. Dis..

[30] Mahmud N., Asch D.A., Sung J., Reitz C., Coniglio M.S., McDonald C., Mehta S.J. (2021). Effect of text messaging on bowel preparation and appointment attendance for outpatient colonoscopy: A randomized clinical trial. JAMA Netw. Open.

[31] Back S.Y., Kim H.G., Ahn E.M., Park S., Jeon S.R., Im H.H., Cho J.H. (2018). Impact of patient audiovisual re-education via a smartphone on the quality of bowel preparation before colonoscopy: A single-blinded randomized study. Gastrointest. Endosc..

[32] Tae J.W., Lee J.C., Hong S.J., Han J.P., Lee Y.H., Chung J.H., Lee M.S. (2012). Impact of patient education with cartoon visual aids on the quality of bowel preparation for colonoscopy. Gastrointest. Endosc..

[33] Calderwood A.H., Lai E.J., Fix O.K., Jacobson B.C. (2011). An endoscopist-blinded, randomized, controlled trial of a simple visual aid to improve bowel preparation for screening colonoscopy. Gastrointest. Endosc..

[34] Guardiola-Arévalo A., Granja Navacerrada A., García-Alonso F.J., Bernal Checa P., Piqué Becerra R., Guerra I., Bermejo F. (2019). Randomized clinical trial evaluating the effect of a visual educational leaflet on the preparation of colonoscopies in hospitalized patients. Rev. Esp. Enfermedades Dig..

[35] Losurdo G., Martino M.L., De Bellis M., Celiberto F., Rizzi S., Principi M., Ierardi E., Iannone A., Di Leo A. (2023). Effect of Visual Booklets to Improve Bowel Preparation in Colonoscopy: Systematic Review with Meta-Analysis. J. Clin. Med..

[36] Ho L.H., Montealegre J.R., Jibaja-Weiss M.L., Suarez M.G. (2019). Impact of colonoscopy preparation video on boston bowel preparation scale score. Gastroenterol. Nurs..

[37] Prakash S.R., Verma S., McGowan J., Smith B.E., Shroff A., Gibson G.H., Mohanty S.R. (2013). Improving the quality of colonoscopy bowel preparation using an educational video. Can. J. Gastroenterol. Hepatol..

[38] Pillai A., Menon R., Oustecky D., Ahmad A. (2018). Educational colonoscopy video enhances bowel preparation quality and comprehension in an inner city population. J. Clin. Gastroenterol..

[39] Archer T., Corfe B., Dear K., Cole A., Foley S., Andreyev H.J.N., EBOPS Study Group (2024). Can an educational video improve the adequacy of bowel preparation for patients undergoing their first colonoscopy? Results of the EBOPS RCT. Endosc. Int. Open.

[40] Hayat U., Lee P.J., Lopez R., Vargo J.J., Rizk M.K. (2016). Online educational video improves bowel preparation and reduces the need for repeat colonoscopy within three years. Am. J. Med..

[41] Ding Y., Vandeleur A., Chinchilla G., Littlemore K., Hodgson R., Rahman T. (2024). Online patient endoscopy education platform improves outpatient bowel preparation quality: Retrospective observational study. Endosc. Int. Open.

[42] Hsueh F.C., Wang H.C., Sun C.A., Tseng C.C., Han T.C., Hsiao S.M., Yang T. (2014). The effect of different patient education methods on quality of bowel cleanliness in outpatients receiving colonoscopy examination. Appl. Nurs. Res..

[43] Ye Z., Chen J., Xuan Z., Gao M., Yang H. (2020). Educational video improves bowel preparation in patients undergoing colonoscopy: A systematic review and meta-analysis. Ann. Palliat. Med..

[44] Rice S.C., Higginbotham T., Dean M.J., Slaughter J.C., Yachimski P.S., Obstein K.L. (2016). Video on diet before outpatient colonoscopy does not improve quality of bowel preparation: A prospective, randomized, controlled trial. Off. J. Am. Coll. Gastroenterol.|ACG.

[45] Walker T.B., Hengehold T.A., Garza K., Rogers B.D., Early D. (2022). An interactive video educational tool does not improve the quality of bowel preparation for colonoscopy: A randomized controlled study. Dig. Dis. Sci..

[46] Dikkanoğlu B., Duman A.E., Hülagü S. (2021). Effect of physician provided education on the quality of bowel preparation. Acta Gastroenterol. Belg..

[47] Shieh T.Y., Chen M.J., Chang C.W., Hung C.Y., Hu K.C., Kuo Y.C., Wang H.Y. (2013). Effect of physician-delivered patient education on the quality of bowel preparation for screening colonoscopy. Gastroenterol. Res. Pract..

[48] Elvas L., Brito D., Areia M., Carvalho R., Alves S., Saraiva S., Cadime A.T. (2017). Impact of personalised patient education on bowel preparation for colonoscopy: Prospective randomised controlled trial. GE-Port. J. Gastroenterol..

[49] Abuksis G., Mor M., Segal N., Shemesh I., Morad I., Plaut S., Niv Y. (2001). A patient education program is cost-effective for preventing failure of endoscopic procedures in a gastroenterology department. Off. J. Am. Coll. Gastroenterol.|ACG.

[50] Liu X., Luo H., Zhang L., Leung F.W., Liu Z., Wang X., Guo X. (2014). Telephone-based re-education on the day before colonoscopy improves the quality of bowel preparation and the polyp detection rate: A prospective, colonoscopist-blinded, randomised, controlled study. Gut.

[51] He X., Lei X., Li J., Li P. (2023). Telephone instructions improve the quality of bowel preparation for colonoscopy: A meta-analysis of randomized controlled trials. PLoS ONE.

[52] Hu C.J., Jiang L.Y., Sun L.Y., Hu C.Y., Shi K.M., Bao Z.F., Zhou F., Xu L., Wang W.H. (2021). Impact of a Telephone Intervention on Bowel Preparation Quality for Colonoscopy in the Elderly. Gastroenterol. Nurs..

[53] Price M., Yuen E.K., Goetter E.M., Herbert J.D., Forman E.M., Acierno R., Ruggiero K.J. (2014). mHealth: A mechanism to deliver more accessible, more effective mental health care. Clin. Psychol. Psychother..

[54] Kang X., Zhao L., Leung F., Luo H., Wang L., Wu J., Guo X. (2016). Delivery of instructions via mobile social media app increases quality of bowel preparation. Clin. Gastroenterol. Hepatol..

[55] Wen J., Feng J., Liu C., Yang D., Zhang Y., Lu N., Huang J. (2022). Increased quality of bowel preparation via smartphone WeChat application: A multicenter randomized controlled trial. Videosurgery Other Miniinvasive Tech..

[56] Lorenzo-Zúñiga V., Moreno de Vega V., Marín I., Barberá M., Boix J. (2015). Improving the quality of colonoscopy bowel preparation using a smart phone application: A randomized trial. Dig. Endosc..

[57] Cho J., Lee S., Shin J.A., Kim J.H., Lee H.S. (2017). The impact of patient education with a smartphone application on the quality of bowel preparation for screening colonoscopy. Clin. Endosc..

[58] Desai M., Nutalapati V., Bansal A., Buckles D., Bonino J., Olyaee M., Rastogi A. (2019). Use of smartphone applications to improve quality of bowel preparation for colonoscopy: A systematic review and meta-analysis. Endosc. Int. Open.

[59] El Bizri M., El Sheikh M., Lee G.E., Sewitch M.J. (2021). Mobile health technologies supporting colonoscopy preparation: A systematic review and meta-analysis of randomized controlled trials. PLoS ONE.

[60] Dao H.V., Dao Q.V., Lam H.N., Hoang L.B., Nguyen V.T., Nguyen T.T., Van Dao L. (2023). Effectiveness of using a patient education mobile application to improve the quality of bowel preparation: A randomised controlled trial. BMJ Open Gastroenterol..

[61] Sharara A.I., Chalhoub J.M., Beydoun M., Shayto R.H., Chehab H., Harb A.H., Sarkis F.S. (2017). A customized mobile application in colonoscopy preparation: A randomized controlled trial. Clin. Transl. Gastroenterol..

[62] Chen G., Zhao Y., Xie F., Shi W., Yang Y., Yang A., Wu D. (2021). Educating outpatients for bowel preparation before colonoscopy using conventional methods vs virtual reality videos plus conventional methods: A randomized clinical trial. JAMA Netw. Open.

[63] Gwag M., Yoo J. (2024). Development and Effectiveness Evaluation of 360-Degree Virtual Reality-Based Educational Intervention for Adult Patients Undergoing Colonoscopy. Healthcare.

[64] Gandhi K., Tofani C., Sokach C., Patel D., Kastenberg D., Daskalakis C. (2018). Patient characteristics associated with quality of colonoscopy preparation: A systematic review and meta-analysis. Clin. Gastroenterol. Hepatol..

[65] Rex D.K., Imperiale T.F., Latinovich D.R., Bratcher L.L. (2002). Impact of bowel preparation on efficiency and cost of colonoscopy. Am. J. Gastroenterol..

[66] Hassan C., Fuccio L., Bruno M., Pagano N., Spada C., Carrara S., Repici A. (2012). A predictive model identifies patients most likely to have inadequate bowel preparation for colonoscopy. Clin. Gastroenterol. Hepatol..

